# Impfungen als Instrument der Pandemiebewältigung

**DOI:** 10.1007/s00103-022-03626-8

**Published:** 2022-12-05

**Authors:** Eberhard Hildt, Lars Schaade

**Affiliations:** 1 Abt. Virologie, Paul-Ehrlich-Institut – Bundesinstitut für Impfstoffe und biomedizinische Arzneimittel, Paul-Ehrlich-Str. 51–59, 63225 Langen, Deutschland; 2 Zentrum für Biologische Gefahren und Spezielle Pathogene, Robert Koch-Institut, Nordufer 20, 13353 Berlin, Deutschland

Im Verlauf der gegenwärtigen SARS-CoV-2-Pandemie hat sich gezeigt, dass Impfungen gegen COVID-19 ein effektives Werkzeug darstellen, um schwere Verläufe von SARS-CoV-2-Infektionen zu verhindern. Das vorliegende Schwerpunktheft des Bundesgesundheitsblatts zum Thema: „COVID-19 und Public Health: Impfungen“ beleuchtet 2 verschiedene Themenkomplexe im Zusammenhang mit der Impfung gegen COVID-19.

Im ersten Themenkomplex beschreiben 3 Arbeiten verschiedene derzeit zugelassene Impfstoffe, den Weg zur Zulassung sowie die Entwicklung von Impfempfehlungen durch die Ständige Impfkommission (STIKO) im Kontext der SARS-CoV-2-Pandemie. Der zweite Themenkomplex umfasst Arbeiten, die Strukturen zur Impfversorgung wie auch zur Erhebung von Impfquoten und des Monitorings der Impfstoffwirksamkeit beschreiben sowie die Einstellung der Bevölkerung zum bisherigen Verlauf der Pandemie zur Impfung. Auch wird auf das Gesundheitsinformationsverhalten und die Gesundheitskompetenz der Bevölkerung in Hinblick auf die COVID-19-Impfung eingegangen.

Der erste Themenkomplex beginnt mit einem Überblick von *Hildt* über derzeit in der EU (bedingt) zugelassene Impfstoffe. Dies sind 2 mRNA-basierte Impfstoffe (BNT162b2, Comirnaty® und mRNA-1273, Spikevax®), 2 auf einem adenoviralen Vektor basierende Impfstoffe (AZD1222, Vaxzevria® und Ad26.COV2.S, Jcovden®), der Untereinheitenimpfstoff Nuvaxovid® (NVX-CoV2373) und der Inaktivatvirus-Impfstoff VLA2001. Die stoffliche Zusammensetzung, die zugrunde liegenden Antigene und ihre Wirksamkeit werden beschrieben. Daneben wird das Zulassungsverfahren dargestellt und die Besonderheiten beschrieben, die es ermöglicht haben, dass in einer bisher noch nie dagewesenen Schnelligkeit sichere und wirksame Impfstoffe gegen COVID-19 geprüft und zugelassen werden konnten.

Neben der Zulassung ist die Chargenprüfung von essentieller Bedeutung für die Versorgung mit sicheren Impfstoffen. Der Beitrag von *Sediri-Schön et al.* beschreibt die gesetzlichen Grundlagen und europäischen Strukturen, auf deren Basis die Überprüfung und Freigabe erfolgen. Am Beispiel der mRNA-Impfstoffe, einer neuen Plattformtechnologie, für deren Charakterisierung viele Methoden zu entwickeln sind, zeigt der Artikel die enormen Herausforderungen für die Produktprüfung auf, hier innerhalb kurzer Zeit parallel zur Produktentwicklung die Methodik zu etablieren und zu validieren, um so sicherzustellen, dass unmittelbar nach der erfolgten Zulassung auch entsprechend getestete Chargen dieser Impfstoffe für die Impfkampagne zur Verfügung standen. Um diese Herausforderung ermessen zu können, stelle man sich das gegenteilige Szenario vor: Trotz eines äußerst schnellen Zulassungsverfahrens wäre die Versorgung mit Impfstoffen daran gescheitert, dass die Produktprüfung nicht hätte Schritt halten können.

Die Arbeit der STIKO ist von herausragender Bedeutung für die Definition von Bevölkerungsgruppen, die in besonderer Weise von der Impfung profitieren, sowie für die ggf. erforderliche Priorisierung und damit die Anwendung der Impfstoffe und die Durchführung der Impfkampagnen. Aufgabe der STIKO ist die Erarbeitung unabhängiger, evidenzbasierter Impfempfehlungen für Deutschland. Gerade bei der Anwendung von Impfstoffen, die auf neuen Plattformtechnologien basieren, können evidenzbasierte Empfehlungen eine Herausforderung darstellen, da erst die entsprechenden Daten vorliegen und ausgewertet sein müssen. *Vygen-Bonnet et al.* beschreiben in ihrem Beitrag die grundsätzliche Vorgehensweise der STIKO und die im Zusammenhang mit der COVID-19-Pandemie bestehenden Besonderheiten und Herausforderungen. Schließlich mussten unter enormen öffentlichen Erwartungen, einer sich kontinuierlich weiterentwickelnden Datenlage und insbesondere anfänglich sehr begrenzter Impfstoffverfügbarkeit Nutzen-Risiko-Abwägungen evidenzbasiert vorgenommen und Impfempfehlungen ausgesprochen werden.

Im zweiten Themenkomplex „Impfverhalten und Impfinformation“ beschreibt der Beitrag von *Meyer et al.* die praktischen Erfahrungen der Kassenärztlichen Vereinigung Westfalen-Lippe (KVWL) bei der Umsetzung der COVID-19-Impfungen in Nordrhein-Westfalen und speziell im Landesteil Westfalen-Lippe. Der Aufbau von Impfzentren gemeinsam mit den Gebietskörperschaften im Auftrag des Landesgesundheitsministeriums und die Durchführung von Massenimpfungen werden dargestellt. Schwierigkeiten ergaben sich vor allem aus der unsteten Verfügbarkeit der Impfstoffe und der Umsetzung der Priorisierung.

Das Fachgebiet Impfprävention des Robert Koch-Instituts (RKI) hat in der Pandemie unter anderem die Erhebung von Impfquoten und das Monitoring der Wirksamkeit der Impfung durchgeführt. Im Artikel von *Siedler et al.* wird ein Überblick über die etablierten Strukturen, Datengrundlagen und Studien gegeben. Das digitale Impfquotenmonitoring (DIM) wird z. B. für die tagesaktuelle Berechnung der COVID-19-Impfquote in mehreren Altersgruppen verwendet sowie für die Erhebung der Inzidenzen nach Impfstatus und die Abschätzung der Impfeffektivität gegen verschiedene Endpunkte (Hospitalisierung, intensivstationäre Betreuung, Tod). Aktuell wird zudem gemeinsam mit dem Paul-Ehrlich-Institut (PEI) die krankenhausbasierte Fall-Kontrollstudie COViK durchgeführt, deren Zwischenergebnisse zur Impfeffektivität in der Delta-Welle vorgestellt werden.

Der Beitrag von *Brandstetter et al.* widmet sich der COVID-19-Impfintention von Eltern bezogen auf ihre Kinder von der ersten zur zweiten Pandemiewelle – vor der Verfügbarkeit des Impfstoffs. Determinanten der Impfintention wurden im Rahmen der „KUNO Kids Gesundheitsstudie“ analysiert. Es zeigte sich, dass die Intention, das eigene Kind impfen zu lassen, insgesamt nur mäßig ausgeprägt war und zur zweiten Welle weiter abnahm. Einstellungs- und kompetenzbezogene Determinanten erwiesen sich als relevant und könnten in einer Impfkampagne gezielt berücksichtigt werden.

Mit dem Gesundheitsinformationsverhalten und der Gesundheitskompetenz der Bevölkerung bezüglich der COVID-19-Impfung befasst sich der Beitrag von *Bosle et al*. Dazu werden die Befunde der dritten repräsentativen Bevölkerungsbefragung Ende 2021 im Rahmen der CoSiD-Studie (Corona-Schutzimpfung in Deutschland) vorgestellt. Untersucht wurden Zusammenhänge zwischen Informationsverhalten bzw. subjektiven Gesundheitskompetenzen und Impfstatus bzw. Impfabsicht sowie zwischen soziodemographischen Merkmalen und subjektiven Gesundheitskompetenzen. Die Autorinnen und Autoren kommen zu Schlussfolgerungen, wie künftige Kommunikationsmaßnahmen gestaltet werden sollten, um Gesundheitskompetenzen hinsichtlich der COVID-19-Impfung zu erhöhen.

Dieses Themenhaft macht deutlich, welche beeindruckenden Leistungen Wissenschaft, Gesundheitswesen und Gesellschaft gemeinsam erbracht haben, um Impfstoffe zur Anwendung zu bringen, die durch ihren guten Schutz vor schweren COVID-19-Verläufen einen wesentlichen Baustein in der Pandemiebewältigung darstellen. Gleichzeitig wird auf bestehende Herausforderungen und Verbesserungsmöglichkeiten hingewiesen.

Wir wünschen Ihnen eine gewinnbringende Lektüre.

